# Hand-Held Tidal Breathing Nasal Nitric Oxide Measurement – A Promising Targeted Case-Finding Tool for the Diagnosis of Primary Ciliary Dyskinesia

**DOI:** 10.1371/journal.pone.0057262

**Published:** 2013-02-20

**Authors:** June Kehlet Marthin, Kim Gjerum Nielsen

**Affiliations:** Pediatric Pulmonary Service, Department of Pediatrics and Adolescent Medicine, Copenhagen University Hospital, Rigshospitalet, Copenhagen, Denmark; Pulmonary Research Institute at LungClinic Grosshansdorf, United States of America

## Abstract

**Background:**

Nasal nitric oxide (nNO) measurement is an established first line test in the work-up for primary ciliary dyskinesia (PCD). Tidal breathing nNO (TB-nNO) measurements require minimal cooperation and are potentially useful even in young children. Hand-held NO devices are becoming increasingly widespread for asthma management. Therefore, we chose to assess whether hand-held TB-nNO measurements reliably discriminate between PCD, and Healthy Subjects (HS) and included Cystic Fibrosis (CF) patients as a disease control group known to have intermediate nNO levels.

**Methods:**

In this cross sectional, single centre, single occasion, proof-of-concept study in children and adults with PCD and CF, and in HS we compared feasibility, success rates, discriminatory capacity, repeatability and agreement between a hand-held electrochemical device equipped with a nNO software application sampling at flow rates 2 ml/s or 5 ml/s, and two stationary chemiluminescence devices, applying both tidal breathing and velum closure techniques.

**Results:**

Measurements were done in 16 PCD patients, 21 patients with CF and 20 HS aged between 3.8 and 60.9 years. Hand-held TB-nNO showed high success rate (96.5–100%) vs. velum closure nNO techniques (70.2–89.5%). Hand-held TB-nNO sampling at flow rate 5 ml/s showed equally high discriminative power (PCD vs. HS [p<0.0001] and PCD vs. CF [p<0.0001]) and reaching close to 100% sensitivity and specificity, superior repeatability (CV% = 10%) and equal limits of agreement compared to TB-nNO by stationary devices and even compared to velum closure sampling.

**Conclusion:**

Hand-held TB-nNO discriminates significantly between PCD, CF and HS and shows promising potential as a widespread targeted case-finding tool for PCD, although further studies are warranted before implementation.

## Introduction

Early diagnosis of Primary Ciliary Dyskinesia (PCD) is important to obtain best possible general care and improve or stabilise lung function after diagnosis and initiation of treatment[1]. Nasal Nitric Oxide (nNO) concentration discriminates significantly between PCD and non-PCD[2]-[6], is quick, non-invasive, and since 2009 recommended in Europe as the preferred first-line test for PCD in advance of confirmatory diagnostic tests[7],[8].

The current recommended method since 2005 is aspiration at constant flow rate from one nostril with gas entrained via the other nostril during velum closure (VC)[9], which may not be compatible with measurements in very young children[7].

Tidal breathing nNO (TB-nNO) measurement is an alternative method that does not include VC. Provided acceptance of the nasal probe, TB-nNO is easier to perform in all age groups since normal breathing is allowed during sampling. We recently reported significant discrimination between PCD and non-PCD (including CF patients) and acceptability of 95% in subjects <6 years of age, with a minimum age for compliance of 14 days of age by TB-nNO technique, using a stationary chemiluminescence device[5].

FeNO measurements using both hand-held electrochemical devices and stationary chemiluminescence devices have previously shown excellent correlation in patients with asthma[10]. Recent introduction of equipment fitted also for nNO measurements by hand-held devices, has brought a new perspective to nNO-measurements as work-up tool in PCD. The electrochemical devices for hand-held nNO measurements are portable, simple to use, and at a markedly lower cost in comparison to the conventional stationary chemiluminescence-based nNO–analysers, and thereby affordable even for small, secondary paediatric centres, with a view to provide early targeted case finding in young children with symptoms suspicious of PCD.

Studies comparing nNO measured by hand-held devices as opposed to stationary analysers reported excellent agreement between hand-held and stationary nNO values in both healthy subjects and patients with allergic rhinitis during single-breath exhalation through face mask[11], and in patients with PCD and CF during both silent and humming exhalation through face mask[12]. Until now, no studies have evaluated hand-held TB-nNO.

We hypothesised that TB-nNO measurement would demonstrate higher feasibility compared to VC-nNO measurement, and further that hand-held TB-nNO values would exhibit equally high discrimination between PCDs and non-PCDs (including CF patients) compared to VC-nNO and to TB-nNO measured by stationary devices.

In this proof-of-concept study we aimed to investigate the discriminative power of hand-held TB-nNO measurements to separate between PCD, CF and healthy and to assess the agreement between hand-held TB-nNO and stationary TB-nNO, and VC-nNO values in children and adults.

## Methods

### Study Design

Prospective, cross sectional, between group and method comparison, single occasion and single centre, and investigator initiated study with no direct or indirect sponsorship from manufacturers of equipment. Data were collected from October 2011 to February 2012.

### Participants

Three groups of highly selected subjects, children and adults, were included: Definite PCDs from the National Danish PCD cohort in which CF and immunodeficiency had been excluded[1]. Diagnosis was based on characteristic clinical symptoms in combination with abnormal low nNO level, at least twice repeated high speed video-microscopic recordings showing abnormal ciliary beat pattern and frequency, and transmission electron microscopy analysis of at least 100 cross sections of ciliary ultrastructure per patient, showing abnormalities according to guidelines[7]. Additionally, measurement of Pulmonary Radioaerosol Mucociliary Clearance (PRMC)[13], was performed in a fraction of patients. CF patients documented by sweat and genotype testing were recruited from CF Centre Copenhagen, Denmark. Non-smoking, non-atopic HS were recruited among siblings, staff members and children of staff.

Subjects with acute respiratory infection within previous 2 weeks were excluded. No concurrent local nasal medication was allowed.

All participants or their parents or guardians gave their written informed consent prior to participation. The study was approved by the local ethics committee of The Capital Region of Denmark (KF 01-045/04).

### Equipment for nNO measurement

Three different equipments were used: hand-held NIOX MINO^®^ Nasal (Aerocrine AB, Solna, Sweden) and to our experience the two most frequently used stationary devices; NIOX^®^ Flex (Aerocrine AB, Solna, Sweden) and CLD 88sp NO analyser (ECO MEDICS^®^ AG, Duernten, Switzerland).

NIOX MINO^®^ Nasal is delivered from the manufacturer for research use with optional sampling rates of either 2 or 5 ml/s. It employs an electrochemical sensor and the collected sample is buffered prior to analysis. The measurement range is 5 to 1 700 parts per billion (ppb). The device returns the result of the buffered analysis as one output on screen after either 2 minutes (sampling rate 2 ml/s [MINO2]) or 45 seconds (sampling rate 5 ml/s [MINO5]). A successful test relies on completed, uninterrupted, sampling throughout the required sampling time.

Both stationary analysers use chemiluminescence, with online measurement and real time display of result on screen, and a sampling rate of 5 ml/s (NIOX^®^ Flex) or 5.5 ml/s (CLD 88sp NO analyser).

### nNO breathing modalities and sampling

All measurements were done in all subjects at one single occasion, within 30 minutes and by one un-blinded, experienced observer. Seven combinations of breathing modalities and analysers were applied: hand-held TB-nNO using 2 different sampling rates, TB-nNO by each stationary nNO analyser, hand-held VC-nNO using exclusively sampling rate of 5 ml/s (impossible with 2 ml/s), and VC-nNO by each stationary analyser. All participants were expected to have triplet data set from each of the seven modalities, i.e. in total 21 expected measurements.

A nasal olive probe with a central lumen connected to the NO-analyser was inserted into one nostril. Patients were interviewed about known anatomical defects or previous nasal surgery, and in such cases the affected side was avoided and the opposite nostril chosen for measurements.

TB-nNO sampling is a non-VC procedure applying normal relaxed tidal breathing during sampling[5]. TB-nNO measurements were performed using four different sampling modalities: hand-held nNO sampled at flow rates of 2 and 5 ml/s, respectively (MINO2 and MINO5), and by each of the two stationary devices.

In both stationary devices, TB-nNO values were averaged from the highest three distinct visible peak concentrations captured within 30 to 40 seconds and read directly as point values on screen[5].

VC-nNO sampling was done both by 1) nasal aspiration technique in which nasal air was sampled continuously with a constant transnasal flow (CLD 88sp NO analyser) in alignment with recommendations[9], and 2) passive aspiration of gas from one nostril during Breath Hold (BH) (NIOX Flex and MINO5). The stationary devices did not require fixed sampling time, but are dependent on time to reach a stable plateau of minimum 4–5 seconds of duration[5]. Sampling duration was typically 20 to 30 seconds, never exceeding 40 seconds.

VC-nNO by MINO2 was not possible due to the fixed sampling duration of 2 minutes, which was incompatible with BH technique.

### Statistical analysis

Median, range, mean and SEM were used for characteristics whenever appropriate. Receiver Operating Characteristic curves were used to determine specificity, sensitivity and cut-off values between groups. Unpaired t-test was used for comparison of mean TB-nNO and VC-nNO values between groups. Reliability of each sampling modality was evaluated as coefficient of variation (CV%) for all subjects. Bland-Altman plots[14], were applied to determine Limits of Agreement (LoA) in TB-nNO measurements. NIOX Flex was used as reference method since TB-nNO results have been previously published[5]. From experience with TB-nNO measurements[5], the hypothesized difference between PCDs, CF and HS would be detected with 95% power with 10 subjects in each group. However, since TB-nNO using hand-held device is largely unexplored, a sample size of 20 per group was chosen. A p-value <0.05 defined the level of statistical significance. Analyses were done using MedCalc© (Version 12.3.0., MedCalc Software, Mariakerke, Belgium).

## Results

### Participants

In total, 57 subjects (18 males) aged between 3.8 and 60.9 years were enrolled: 16 definite PCDs, 21 CF patients, and 20 HS. Subject characteristics, results of diagnostic tests and respiratory infections in participating CF patients and patients with PCD are shown in Table 1.

**Table 1:** Characteristics, diagnostic tests and respiratory infections in participating subjects, cystic fibrosis patients and patients with primary ciliary dyskinesia.

Characteristics	Healthy subjects	Cystic Fibrosis	Primary Ciliary Dyskinesia
Number	20	21	16
Age, median, years	31.0	11.0	25.9
(range)	(15.6–58.4)	(3.9–23.2)	(8.4–60.9)
Gender, M:F	4∶16	10∶11	4∶12
Smoking	Non-smokers	Non-smokers	Non-smokers
Diagnostic tests			
*Ciliary Beat Pattern and Frequency*	irr	irr	
Normal frequency, asynchrony			1
Low frequency, asynchrony			11
Immotility			3
Normal			0
Inconclusive			1
*Ultrastructural Defect by EM*	irr	irr	
Outer Dynein Arm			6
Inner Dynein Arm			1
Outer and Inner Dynein Arm			2
Radial Spoke			2
Central Pair			1
Hydin[19]			1
Peripheral Microtubuli			1
Inconclusive			2
*PRMC* §			9 out of 11
*CFTR-mutation*	irr		irr
ΔF508 homozygous, n		12	
ΔF508 compound (n)		*W1282X (2), N1303K (2), R1066C (1), 3849+ 1G- >A (1), 1717-g1 (1), del exon 2- 3 (1), 3659delC (1)*	
Respiratory infection			
None, n	irr	11	10
Chronic infection^#^, Pathogen (n)		*P. aeruginosa (1), A. xylosoxidans (2), S. maltophilia (1)*	*P. aeruginosa (2)*
Actual pathogen at day of visit, single isolates or combinations presented (n)		*S. aureus (2), A. Fumigatus (1), S. aureus + H. influenzae + M. catharhalis (1), S. Maltophilia (2), S. aureus + M. catharhalis (1), S. aureus + P. Aeruginosa* + *A. fumigatus (1)*	*A. fumigatus (1), H. influenzae (1), S. aureus + H. influenzae (1)*

§ : Pulmonary Radioaerosol Mucociliary Clearance – number of abnormal results in number of tested patients – 2 out of 11 were inconclusive. #:Chronic infection defined when bacteria were isolated in more than 50% of respiratory cultures within the last year. *P. aeruginosa: Pseudomonas aeruginosa, A. xylosoxidans: Achromobacter xylosoxidans, S. maltophilia: Stenotrophomonas maltophilia, S. aureus: Staphylococus aureus, A. Fumigatus: Aspergillus fumigatus, H. Influenzae: Haemophilus influenzae, M. Catharhalis: Morexella catharhalis.* Irr: Irrelevant.

### Ambient NO concentration

Mean (SD) ambient NO during measurements was 9.9 ppb (11.6 ppb) measured by the CLD 88sp NO analyser and 9.0 ppb (11.7 ppb) measured by NIOX Flex analyser. NIOX MINO does not provide an ambient NO measure.

### Feasibility and success rate

The total number of single nNO measurements in this study reached 1.096. Feasibility and rate of successful measurements by each modality is given in Table 2. TB-nNO modalities showed higher success rates (96.5 to 100%) than VC-nNO techniques (70.2 to 89.5%). The success rate of TB-nNO by MINO5 and stationary analysers was equal (100%). In the study 3 out of 57 subjects were children < 6 years of age, all cooperated to TB-nNO by MINO5. The age of subjects with unsuccessful measurements by VC-sampling ranged from 3.9 to 43.6 years and unsuccessful measurements, including lack of triple measurements, were much more prevalent among both CF and PCD patients as compared to HS (Table 2). Young children, in particular, found it difficult to complete full 2 minutes of TB-nNO sampling required by MINO2.

**Table 2:** Success rates and feasibility of nasal Nitric Oxide sampling during tidal breathing and velum closure modality, using hand-held and stationary analysers in patients with primary ciliary dyskinesia, cystic fibrosis and in healthy subjects.

Sampling and breathing modality	Success rate	Unsuccessful measurements	Subjects not achieving triple data
	N (%)	Median (range) age	Number, %
		(n; diagnosis)	(Age, diagnosis of subjects)
**HAND-HELD ANALYSER**			
MINO5, TB (nVC)	57/57 (100%)	-	2/57, 3.5%
			(8y, PCD), (41y, HS)
MINO2, TB (nVC)	55/57 (96.5%)	4y & 7y	2/55, 3.6%
		(2; CF)	(4y, CF), (5y, CF)
MINO5, BH (VC)	40/57 (70.2%)	8.7y (3.9y to 43.6y)	3/40, 7.5%
		(13; CF) (4; PCD)	(19y & 61y, PCD) (22y, HS)
**STATIONARY ANALYSER**			
NIOX FLEX, TB (nVC)	57/57 (100%)	-	1/57, 1.8%
			(8y, CF)
CLD 88sp, TB (nVC)	57/57 (100%)	-	1/57, 1.8%
			(22y, CF)
NIOX FLEX, BH (VC)	49/57 (86%)	8y (4y to 16y)	1/49, 2.0%
		(7; CF) (1; PCD)	(16y, PCD)
CLD 88sp^§^, (VC)	51/57 (89.5%)	7y (4y to 9y)	-
		(5; CF) (1; PCD)	

Hand-held nNO was measured by sampling rate of both 2 ml/s (MINO2) and 5 ml/s (MINO5). Two stationary analysers (CLD 88sp and NIOX Flex) were employed. Both non-velum closure (nVC) and velum closure (VC) results are shown. §: VC accomplished by standard manoeuvre during exhalation against resistance in a mouthpiece. The fraction of cooperative measurements and according success rates are shown for each test modality, with age and diagnoses given for uncooperative individuals. TB: Tidal Breathing, BH: Breath Hold, PCD: Primary Ciliary Dyskinesia, CF: Cystic Fibrosis, HS: Healthy Subjects.

### Discriminative capacity

**Figure 1:**
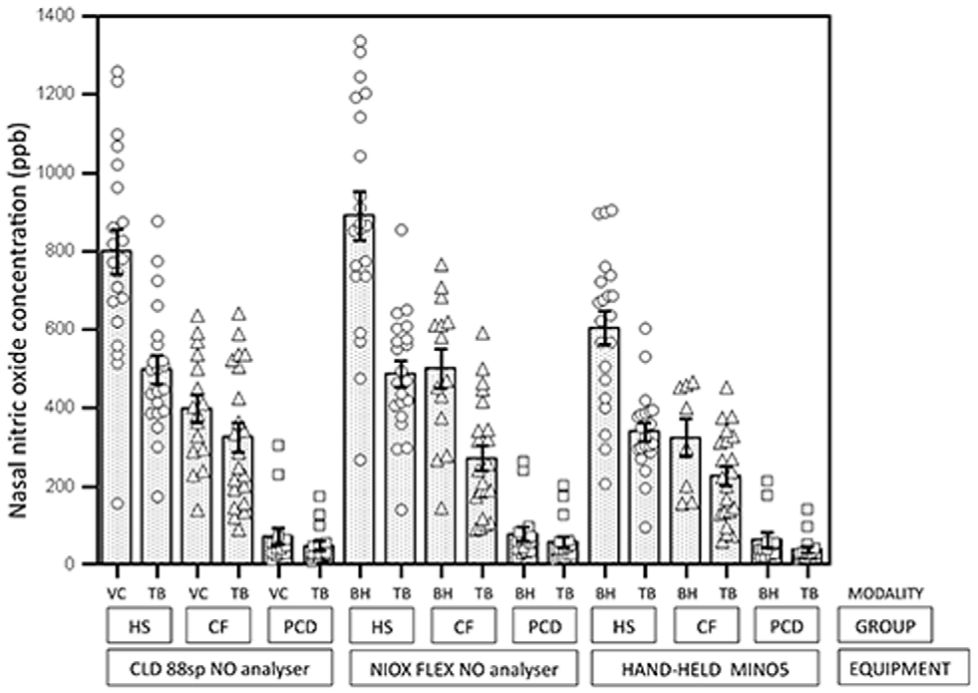
Nasal nitric oxide concentration measured by hand-held and stationary nitric oxide analysers during velum closure, breath hold and tidal breathing in healthy subjects, patients with CF and patients with PCD. Legend: all measurements are displayed as open point markers and bars designate mean nNO concentrations (ppb) by each modality in each subject group. Thin black bars show ±1 SEM. Healthy Subjects (HS) are shown as open circles, Cystic Fibrosis (CF) is shown as open triangles and Primary Ciliary Dyskinesia (PCD) is shown as open squares. VC: Velum Closure, TB: Tidal Breathing, BH: Breath Hold, ppb: parts per billion.

nNO discriminated highly significantly between PCD and HS and between PCD and CF irrespective of sampling and breathing modality (Figure 1, Table 3). Hand-held TB-nNO (MINO2 and MINO5) showed equally high discriminative power compared to TB-nNO by stationary analysers and even compared to VC-sampling. Hand-held nNO levels were overall lower than levels measured with stationary analysers. TB-nNO levels were markedly lower than VC- and BH-nNO primarily in HS and less so in CF patients, while such differences were not seen between neither equipment nor modality of breathing and sampling in PCDs (Fig 1, Table 3).

**Table 3:** Discrimination as reflected by mean nasal Nitric Oxide concentrations in parts per billion during tidal breathing and velum closure modality, using hand-held and stationary analysers in patients with primary ciliary dyskinesia, cystic fibrosis and in healthy subjects.

Sampling and breathing modality	Healthy subjects	Cystic Fibrosis	Primary Ciliary Dyskinesia	p-value *	p-value **
	Mean ± SE	Mean ± SE	Mean ± SE		
	(n)	(n)	(n)		
**HAND-HELD ANALYSER**					
MINO5, TB (nVC)	340±23	226±25	38±34	<0.0001	<0.0001
	(21)	(21)	(16)		
MINO2, TB (nVC)	752±59	490±73	74±23	<0.0001	<0.0001
	(21)	(19)	(16)		
NIOX MINO5, BH (VC)	603±42	324±49	64±18	<0.0001	<0.001
	(21)	(8)	(12)		
**STATIONARY ANALYSER**					
NIOX FLEX, TB (nVC)	486±34	273±32	59±14	<0.0001	<0.0001
	(21)	(21)	(16)		
CLD 88sp, TB (nVC)	499±35	326±38	48±12	<0.0001	<0.0001
	(21)	(21)	(16)		
NIOX FLEX, BH (VC)	890±62	501±49	79±19	<0.0001	<0.0001
	(21)	(14)	(15)		
CLD 88sp^§^ (VC)	799±57	399±36	72±21	<0.0001	<0.0001
	(21)	(16)	(15)		

Hand-held nNO was measured by sampling rate of both 2 ml/s (MINO2) and 5 ml/s (MINO5). Two stationary analysers (CLD 88 sp and NIOX Flex) were employed. Both non-velum closure (nVC) and velum closure (VC) results are shown. §: VC accomplished by standard manoeuvre during exhalation against resistance in a mouthpiece. TB: Tidal Breathing, BH: Breath Hold. *: Primary Ciliary Dyskinesia versus Healthy Subjects; **: Primary Ciliary Dyskinesia versus Cystic Fibrosis.

### Cut-off values

Cut-off values, sensitivity and specificity are given in Table 4. Different sampling methods provided different cut-off values. Sensitivity was 100% by all methods, thereby leaving no false negatives with the given cut-off values. Specificity was 100% for TB-nNO by MINO5 and hence comparable to the stationary analysers. TB-nNO by MINO2 showed markedly lower specificity compared to all other modalities. Area under Receiver Operating Characteristic curves (not shown) were all 1 except for TB-nNO by MINO2 (AUC = 0.991).

**Table 4:** Cut-off values, sensitivity and specificity in the discrimination between patients with primary ciliary dyskinesia and healthy subjects by nasal Nitric Oxide concentrations (ppb), during tidal breathing and velum closure modality using hand-held and stationary nNO analysers.

Sampling and breathing modality	Cut-off (ppb)	Sensitivity (%)	Specificity (%)
**HAND-HELD ANALYSER**			
MINO5, TB (nVC)	142	100	100
MINO2, TB (nVC)	362	100	90.5
MINO5, BH (VC)	214	100	95.2
**STATIONARY ANALYSER**			
NIOX FLEX, TB (nVC)	202	100	95.2
CLD 88sp, TB (nVC)	175	100	95.2
NIOX FLEX, BH (VC)	262	100	100
CLD 88sp^§^ (VC)	303	100	95.2

Hand-held nNO was measured by sampling rate of both 2 ml/s (MINO2) and 5 ml/s (MINO5). Two stationary analysers (CLD 88 sp and NIOX Flex) were employed. Both non-velum closure (nVC) and velum closure (VC) results are shown. §: VC accomplished by standard manoeuvre during exhalation against resistance in a mouthpiece. Cut-off values (ppb) with associated sensitivity (%) and specificity (%) were calculated from Receiver Operating Characteristic analyses.

BH: Breath Hold, TB: Tidal Breathing, VC: Velum Closure, nVC: non-Velum Closure.

### Repeatability

Repeatability within three measurements in the same subject was in favor of MINO5 having a CV% of 10%, followed by the stationary devices; NIOX Flex: 12.3%, CLD 88sp: 12.7%. The repeatability of MINO2 was poor (CV% 19.3%).

### Agreement between hand-held and stationary TB-nNO measurements

The ranking order of LoA (mean difference) using NIOX Flex as the reference method was: CLD 88sp with LoA of −22.6 to 44.1 ppb, MINO5 with LoA of −43.0 to 84.7 ppb, and MINO2 with LoA of −116.9 to 86.8 ppb.

## Discussion

This is the first proof-of-concept study comparing measurements of TB-nNO between stationary[5] and hand-held analysers. NIOX MINO® Nasal is, to our knowledge, currently one of very few commercially available hand-held devices, but so far no guidelines exist for their use in PCD work-up. Non-VC-nNO measurement such as TB-nNO technique is easy to perform even in young children[5] and has previously been found to discriminate significantly between PCDs and HS and between PCD and CF using stationary nNO analysers[5],[15].

In this study, hand-held TB-nNO measured at a flow rate of 5 ml/s showed very promising results, was simple to perform with high feasibility and high success rate even in the youngest children, and exhibited excellent discriminative capacity between HS and PCDs, and between PCD and CF. Discrimination was comparable to TB-nNO measurements using stationary analysers and in concordance with published TB-nNO results using stationary equipment[5],[15]. The discriminative power of TB-nNO measured by MINO5 was comparable to nNO measured by the recommended VC-sampling techniques[9], and in addition with higher success rate and feasibility. Furthermore, MINO5 exhibited superior within-subject repeatability, and comparable agreement with stationary analysers.

MINO5 was superior to MINO2 in terms of success rate (100% vs. 96.5%), false positives (specificity 100% vs. 90.5%), within-subject repeatability (CV% of 10% vs. 19.3%), and agreement with stationary reference method (NIOX Flex). In addition, to our experience, cooperation to 2 minutes of sampling, which is required by MINO2 sampling, was much to difficult for especially the young children. Thus the results of this study strongly suggest that hand-held TB-nNO should be measured at a sampling rate of 5 ml/s, abandoning sampling at 2 ml/s.

As expected, hand-held TB-nNO was more feasible compared to VC-nNO by hand-held equipment in terms of overall success rate including successful measurements down to 3.9 years of age. Moreover, hand-held TB-nNO showed superior success rate to VC-nNO in PCD and CF patients probably because TB-technique is less exhausting.

The concept of using a hand-held device for nNO measurement to discriminate between PCD, CF and HS has already been addressed by Montella and coworkers[12]. However, they employed a substantially different and more demanding method, not likely feasible in young children. They did assessments by nasal aspiration/insufflation via one nostril or by nasal silent exhalation through a facemask and also during humming and found it as effective as the stationary analyzer for assessing nNO during silent and humming exhalation.

A simple primary test is definitely needed in the attempt to achieve early diagnosis in children with PCD, since secondary confirmatory tests (ciliary beat pattern and frequency analysis together with evaluation of ciliary ultrastructure) are time consuming and often require repeated unpleasant nasal brushings or scrapings. The motivation for introducing hand-held nNO measurement, as first-in-line tool in the diagnostic work-up for PCD is the markedly lower cost compared to conventional stationary analysers, which make them affordable even to small PCD centres and secondary paediatric centres. Secondly, they are very simple to use with need of minimal training of staff, and with minute demand of maintenance and lack of need for calibration.

A recently published questionnaire survey including 194 centres (26 countries) handling PCD patients across Europe, disclosed that only 46% used nNO as the primary test in PCD work-up, whereas as many as 36% used the saccharin test[16] despite the latter being obsolete and abandoned in international recommendations[7]. The explanation for choosing saccharin test over nNO could be well explained both by the high cost of stationary nNO equipment and the high maintenance-costs. The same survey showed that PCD care is widely decentralised in Europe, with an average number of only four PCD patients per centre and with the need for smaller centres to refer patients to larger centres for diagnostic work-up[16]. For such small centres, a more widespread use of nNO may be enhanced by the current availability of cheap hand-held nNO-analysers. Furthermore, it is assumed that hand-held FeNO measurements, now widely recommended in certain aspects of asthma management[17], are already becoming increasingly implemented in pulmonary and paediatric centres across Europe and US. This will likely pave the way for the additional and optional tool for nNO measurements.

### Limitations of hand-held equipment

There are some limitations to the hand-held analyser used in this study, since e.g. even short interruptions during sampling result in measurement error. Hence, sniffing and crying may require multiple attempts (>3) or even make measurements impossible in an unwilling child. Moreover, especially the 2 full minutes of required TB-nNO sampling by MINO2, made cooperation difficult in the youngest children.

Furthermore, our results clearly demonstrates that TB-nNO measurement as such and by any equipment does not precisely cover the full range of nNO from the very high levels in HS to the extremely low levels in PCDs. Indeed, it seems that precision of TB-nNO, compared to the gold standard using VC-nNO sampling, increases inversely with lower levels of nNO. A phenomenon favouring its use for targeting possible PCDs, but potentially invalidates the use of TB-nNO in patients with e.g. allergic rhinitis.

### Limitations, precautions and future perspectives

In this study, the groups of subjects were highly selected enrolling only definitive PCDs and CF patients diagnosed prior to inclusion. Indeed, the discriminative capacity in a random population of referred patients was not investigated. Furthermore, only few young children were included. Hence, this study provided proof-of-concept for the method as such, but did not provide sufficient data concerning young and none regarding newly referred children. Indeed, in our previous published study of TB-nNO measured using stationary analyser we reported a considerable fraction of false positive cases among newly referred young children[5], which raises questions for future studies. Hand-held TB-nNO applied on a similar less well-defined cohort of mixed referrals for PCD work-up, including a higher number of young children and potentially also infants, might face a similar overlap between groups. Moreover, normal or even high nNO values in PCD has been previously described, although this is rare[3],[5],[18]. With respect to these previous findings, we believe that a high index of clinical suspicion of PCD should always lead to further investigational tests - despite above-cut-off level of nNO.

It must be emphasized that nNO measurements have no place in diagnosis of CF. In this study these measurements only further strengthened the proof-of-concept by demonstrating that hand-held devices completely reflect the intermediate levels of nNO in CF patients (between HS and PCDs), which has been also shown with stationary devices[5].

Hand-held TB-nNO may hold promising potential as targeted case-finding tool for PCD in contrast to a formal screening tool, which is much more comprehensive and currently not realistic with the available methods. Introduction as a primary test in combination with thorough clinical assessment and the very essential history may provide quick assessment and guidance as to either rule out PCD, or refer for confirmatory diagnostic tests in specialised centres. Hence, delay of referral may diminish, and early PCD diagnosis and prevention of missed diagnoses will hopefully come a step closer. However, although it may seem attractive to implement hand-held nNO devices in primary or secondary paediatric centres, some precautions need to be addressed: hand-held TB-nNO needs validation in a real-life scenario among primary and specialised centres with the very heterogeneous population of a large number of referrals with upper and lower respiratory disease involvement. Experiences from such studies may subsequently develop into an algorithm to be used in primary centres to interpret specific levels of nNO values and to determine when to repeat tests, when to refer or when to rule out PCD. Moreover, specific TB-nNO cut-off levels are needed for infants and young children. Finally, optimising the technology behind hand-held nNO could be desirable. From the experience of this study, the ideal nNO hand-held analyser should be simple to use with substantially shorter sampling time requirements and accepting short pauses during measurements, and preferentially also provide real time display of measurement quality.

In conclusion, hand-held TB-nNO discriminates significantly between highly selected groups of PCD, CF and HS, and has equal discriminative power compared to both TB-nNO using stationary analysers and to VC-nNO measurements. Hand-held TB-nNO was in excellent alignment with stationary TB-nNO and more feasible than VC-nNO in both young children and in patients with PCD and CF. TB-nNO measured by sampling rate of 5 ml/s was superior to sampling at 2 ml/s, and we suggest a sampling rate of 5 ml/s to be used in both children and adults. Hand-held TB-nNO exhibits promising potential as a targeted case-finding tool for PCD.
